# Medical Miscellanies

**Published:** 1817-04

**Authors:** 


### MEDICAL MISCELLANIES.

### THE PORTE FEUILLE, No. IV.

OR,

DEPOT FOR DETACHED FACTS AND INTERESTING PHÆNOMENA, OCCURRING IN THE PRACTICE OF THE FRIENDS OF THE MEDICO-CHIRURGICAL JOURNAL.

"Ore trahit quodcunque potest atque addit acervo."

*Case of Injury of the Head, terminating fatally in Seven Hours, with the Appearances on Dissection.*

THE following very illustrative case seems well suited to the general design of the *Porte-Feuille*, and may be thought not unworthy of a place there.—The patient, (a marine) a man of middle age, large, heavy, and athletic, while standing on the gang-way of one of His Majesty's ships, by an unguarded step, slipped and fell backwards into the *waist* or deck below, a height of about seven feet. By evil luck his head had pitched upon a small fragment of a beef bone that happened to be lying on the deck, and this was found to have cut his undress cap, and to have inflicted a small contused wound of the integuments of the head, betwixt the apex of the lambdoidal suture and the occipital ridge. The accident happened a little after one o'clock, P. M. He was stunned by the fall, and carried into the sick-berth in a state of insensibility. I saw him on the instant, and found the pulse thready; respiration faint and slow; face cadaverously pale, and every symptom of diminution, almost amounting to suspension, of the *vires vitæ*. I directed his temples to be chafed with aq. carb. ammoniæ, and some of the concrete salt to be held at intervals to his nose. Meanwhile, the small wound was examined: it reached no deeper than the tendinous aponeurosis of the occipito-frontalis muscle. I soon satisfied myself that no fracture or depression existed. In about ten minutes, the patient recovered his recollection, and the possession of his faculties. He complained much of pain in his head, more particularly in the forehead, and repeatedly applied the palm of his hand to it, with an expression in his countenance of suffering. He now also vomited his dinner, which had been taken an hour before.

By this time his bed was ready, and before laying him down, as the vital functions were now restored, I directed my assistant to take a quantity of blood from his arm, and to follow it up with a purgative. At this moment, being sent for by the captain to report upon the nature of the accident, I went away, and stated to that officer that the patient had been much stunned by the fall, but that he had now recovered his faculties; and as the wound was in itself trifling, I had no great reason to expect any serious consequence from the accident.

I state this circumstance the more particularly, in order to impress how guarded we ever ought to be in injuries of the head, and how apt we are to be led into a wrong prognosis.

In the midst of some desultory conversation that followed upon my report, I was interrupted by my assistant coming to beg I would look again at the patient, as, while his arm was binding up, he had gradually relapsed into a state of stupor, from which he could not be roused. On seeing him, I found it to be too true; and at the time, remarked to my assistant, that the case explained itself: that the first stupor arose evidently from *concussion*, as it is called; but that the second stupor, occurring after an interval of recollection, undoubtedly was caused by *compression* on the brain from extravasated blood, for that no doubt the shock of the fall had ruptured a blood-vessel, besides producing commotion of the general substance of the encephalon." Without an instant's delay, thirty-four ounces of blood were drawn from the arm by a large orifice, the patient being held in the semi-erect posture. This was done about twenty minutes after the fall, but no relief whatever followed. A purging enema was afterwards given.

At three o'clock, no relief having supervened to the bleeding, but the stupor continuing as profound as ever, I thought it *just possible* that depression of the bone might exist, though it could not be discovered by outward examination. I therefore dilated with the scalpel the external wound, and exposed the bone. The pericranium appeared sound and attached, I did not therefore scrape it away.

In making the above incision, a small branch of the right occipital artery was divided, and poured out its blood with considerable rapidity. Deeming an evacuation of this sort highly favourable to my views, I allowed it to bleed till it stopped spontaneously. In this manner sixteen ounces were obtained, but no dawning of returning recollection followed.

About six o'clock, the stupor remained undiminished, and the enema had not operated. The pulse was now rather small and weak, and the aspect of the patient discouraging; but as I firmly believed that evacuations embraced the only remaining hope for his life, I took twenty-eight ounces more of blood from the arm. I would have pushed the depletion even farther, as the man was bulky and very plethoric, but was deterred by a remarkable flagging in the pulse. I determined, therefore, to shave his head, and apply a blister over the whole of it. Time, however, was not allowed for the operation of this remedy, for the patient expired shortly after eight o'clock, little more than two hours from the last bleeding; since which, indeed, his breathing had been gradually growing more stertorous and laborious.

*Dissection.*—Next morning the head was opened; and, as was previously expected, I found four ounces of coagulated blood extravasated betwixt the dura and pia mater, which rolled out in large clots immediately on the former membrane being clipped. This large extravasation was situated under the right temporal bone, and was directly opposed to the site of the external wound. Under the external wound, indeed, no extravasation existed; but in this situation, on examining from within the skull-cap, now removed from the dura mater, I perceived a capillary fissure, nearly an inch in length. I could scarcely insinuate my finger nail betwixt its edges, and therefore was not surprised that I had failed to discover it from without the day before, when I cut down to the bone, as the pericranium was firmly attached all round the fissure. There was no blood or serum extravasated in the ventricles or in the substance of the brain.

Remarks.—Cases like the above are to be found in the works of Pott and others, and the pathology of them is sufficiently understood. The violence of the fall in the above instance had produced not merely stunning, but the rupture of an arterial branch, probably a twig of the meningeal. During the primary stupor, caused by concussion, such was the general prostration of the vital powers, and the feebleness of the heart's action, that no actual effusion of blood took place for a time from the ruptured vessel. But no sooner had the patient recovered his faculties, and the heart's free motion returned, than extravasation began to take place by successive jets; and, in less than ten minutes, became so considerable as, by its mechanical pressure, to produce secondary and permanent stupor.

The extravasation being on the side of the head, almost directly over against the place of the external wound, affords an instance of what has been called *contre-coup*.

A question here presents itself: "Of what importance, in a practical view, would the fissure have been, had it been discovered before death? I think of none at all, as it was entirely capillary, and of course unaccompanied with depression or spiculæ. "Would it have justified the application of the trephine?" I think not, as dissection proved that it could not have directed us to the place of the extravasation: that extravasation, indeed, might have been, with equal likelihood, in the base of the skull, or in the substance of the brain.

It has often been said, that misfortunes seldom come single: accordingly, the very next evening after the above fatal accident, another man fell down the same gang-way, by the main hatch-way, into the *cable-tier*, a height of *sixteen feet*. The stupor was profound and alarming, as in the former case; but by liberal bleeding he recovered his faculties, and in eight days was quite well.

The obvious inference from the retrospect of these two cases is, that we cannot, in any instance, estimate the danger of the injury from the mere height of the patient's fall; and that attention to a circumstance of that kind, if it now and then guides our prognostic, more frequently tends to mislead, and betray us into a wrong one. R.

---

### *Cardiac Disease with Dropsical Effusion, long counteracted by Medicine.*

Early in the year 1815, the Reporter was consulted by a gentleman, forty-five years of age, who had been in valetudinary health for a considerable time. The prominent features of his complaint, at this time, were dyspnœa, especially when walking fast or ascending stairs. His legs and feet were œdematous; the urine scanty, and depositing a lateritious sediment. Ægritudines somni; starts suddenly out of his sleep. Epigastrium full, and tender on the right side over the liver. Thorax sounds badly about the region of the heart; pulse unequal, and irregularly *intermittent;* countenance very sallow and unhealthy. Has slight cough and trifling expectoration, occasionally tinged with blood. Pain from the margins of the left ribs through to the scapula of the same side. Feels some palpitation of the heart on being agitated in mind, or on unusual exertion of body; bowels rather confined, and the secretions dark coloured; feels occasionally a dull pain across the region of the bladder, which he imputes to gravel. Appetite various, but not good. Spirits generally depressed. Much disturbed with flatulence after food; pain in the left arm. Cannot give a very distinct history of his complaint, but says that the foregoing symptoms have been gradually increasing for more than a year.

The blue pill, aloes, squill, and digitalis were given twice a-day, with a saline diuretic medicine. He was enjoined abstinence, in respect to animal food and fermented liquors, and guarded against agitation of mind or exertion of body. In the course of a week, the mouth was tender, and the œdema of the lower extremities subsided as the urine increased. The alvine secretions also became of a better quality; and in three weeks the symptoms were all ameliorated, except the intermissions in the pulse, which never disappeared. He was kept a month on this plan, when the aloes and blue pill, in reduced quantities, were made a kind of permanent medicine with light tonics. Between this time and December 1816, or nearly two years, he had various relapses, and was invariably relieved by the above-mentioned means, followed by light bitters and tonics. The irregularity and intermission of pulse never forsook him, though, in other respects, he often considered himself to be nearly in good health. In the beginning of December, 1816, the œdema of the lower extremities came on suddenly, with the old train of thoracic symptoms, and the cellular membrane of the scrotum became distended to a great size with water; the following diuretic, which had so repeatedly carried off serous effusions before, was also effectual in relieving him this time.—℞. Acidi tartarici gr. xv; carbonatis sodæ exsiccatæ ℈j; spiritus ætheris nitrici ♏xxx. tincturæ scillæ ♏xij; infusi digitalis. aquæ juniperi āā ℥ß. Misce fiat haustus bis terve in die sumendus. He got better, and was at his duty, as usual, during the day at his office. One night, while reading the affecting loss of the Harpooner, he fell down and instantly expired. Leave was not obtained to open the body, but no doubt can be entertained of organic disease of the heart, complicated probably with effusion in the pericardium. I.

---

### *Ulceration of the Pudendum.*

The subject of this disease was a child 16 months old; she was first seen by the Reporter on the 2d of December, 1816, when both labia pudendi were found tumefied, and of a dark copper-like colour: their inner surfaces ulcerated, as were also the nymphæ and clitoris, presenting the appearance of large, spreading, venereal chancres. There was a considerable discharge of thin, fœtid, yellow matter, with brown streaks; the mons veneris appeared swoln; the perinæum and anus were of a glassy red colour, and indicated approaching ulceration. Pulse 90, skin hot and dry, tongue of a yellow brown colour, thirst, countenance of a tallowy appearance, bowels torpid, abdomen soft. The mother stated that the child had been "drooping" for a fortnight, having lost its spirits and appetite; but the commencement of present appearances had only been observed about five days. The treatment directed was this: To wash the parts frequently with tepid liq. plumb. acetat. dilut. and to let folds of linen, wetted with the same, be kept constantly applied to them. Two grains of calomel, and 12 of jalap, were divided into six parts; one of which was given night and morning. Three days subsequent to the employment of these means, with an antiphlogistic regimen, the child was again visited. The labia had lost their dark colour, and the tumefaction, both of them and the mons veneris, had subsided; the appearance of the perinæum and anus much improved; the ulcerations cleaner; discharge more healthy in consistence and colour, and less in quantity; the alvine discharges had been copious, of various colours, slimy and offensive. For the latter 24 hours, the child had not passed any urine, except with the stools, and then apparently with great pain. Half a pint of gruel was given as an injection; a bladder of warm water applied to the pubic region; the wash and powders, before directed, were continued; and a piece of sponge imbued with oil was inserted between the labia. Under this treatment, for three weeks, the child progressively improved, and in 28 days, from the period it was first adopted, she was discharged cured. L.

March 6th, 1817.

[The above appears to be clearly one of those cases described by Mr. Kinder Wood, in the Medico-Chir. Transactions (vide No. 13

of this Journal, p. 25); but the treatment is materially different, inasmuch as our correspondent L did not find it necessary to give bark, wine, and opium, as Mr. Wood has recommended.

In truth, the diseases are rare indeed where the above medicines are necessary in children; even in adults, their use is every day becoming more limited. EDITORS.]

---

*Hernia.*

*February 6th*, 1814. J. N. Merle, seaman, ætat. 30, was received on board this afternoon, labouring under a bubonocele of the left side. At present (now 18 hours since the receipt of the accident) complains of but little pain of tumour, which is large; but suffers much from sharp pains darting across the hypogastric region, with nausea, and constant vomiting; pulse 80, and full; tongue dry in the middle, and covered with a brown fur; bowels constipated. Many attempts to reduce the insestine by the taxis; to have a tobacco clyster; but all proved abortive.

*Six o'clock p. m.* Soon after administering the enema, the pulse became feeble, and a diffused moisture broke out. No fæces had passed; tenesmus and vomiting: to be put into the warm bath.

*Nine o'clock.* All efforts by the taxis unavailing; breathing laborious, and complains of more pain of tumour and lower parts of abdomen; pulse 60, slow. The enema to be repeated.

*February 7th.** *Four o'clock a. m.* Pulse 62, inclining to be full; has slept none, and has passed no fæces; breathing easier; tongue parched; vomiting at intervals. The enema to be repeated, and cold applications to the tumour.

*February 7th. Six o'clock.* The enema he immediately rejected; pulse has risen to 70; at present appears much exhausted, and says he feels himself easy; inclines to sleep.

*Twelve o'clock.* It was now determined to perform the operation, as the only chance of giving him relief. In the mean time he was put into the warm bath, and *two veins* opened at the same instant. Syncope was induced after the loss of about ℔ij ß of blood; the syncope continued for some minutes, when he was placed on a bed in a suitable position, and the gut was easily reduced.

*February 13th.* Dismissed cured, wearing a truss.

Much as has been said for and against bleeding, it appears to me to be attended with little comparative advantage, unless it is carried to the *fainting point*, which may easily be done, in almost every case, with impunity. The abstraction of blood, in every herniary case, may be said to be attended with some benefit, in preventing or checking inflammatory action; and very few cases of this complaint comes before the surgeon where this practice may be contraindicated. M.

---

* Received but a small portion of last clyster, which he immediately voided.

### MISCELLANEOUS.

Quicquid agunt Homines.

### *To the Editors of the Medico-Chirurgical Journal & Review.*

GENTLEMEN,

The late sanguinary war, which separated us from our continental brethren, as much in a literary as in a political and commercial point of view, has evidently shed a most malignant influence on medical science in England. It appears from Professor Sprengel's *concise* retrospect of the last ten years' German medical literature (republished, and republishing in the *Edin. Med. and Surg. Journal)*, that we are here, in this island of fogs, completely in the dark on most, if not all points of *doctrine*, at least in medical matters. It was heart-breaking enough to have the *titles* of those innumerable tomes of precious knowledge spread before us; but to tantalize us, occasionally, with a *morceau*, was too bad of Professor Sprengel, as well as of the English translator. Thus, how dim is the glimmering light which a Brodie has thrown on *secretion*, compared with the effulgence of German sunshine on that subject! "Gradually it became more *evident*, that the *different oxidability* of the layers, of which the solids of the living body consist, developes an *imponderable matter*, which, conducted through the nerves, is consumed in the secretory organs, and by the functions of the muscles." *Ed. Journ.* 394.

How dull and prolix must appear the researches of Hunter, Wilson, Thomson, &c. on inflammation, compared with the luminous discoveries of our brethren on the Continent. Marcus has there shewn that inflammation is— "*the seizing of the electrical moment in the dimensions;*" a theory at once so *evident and convincing*, that I am astonished Sprengel should have thought it necessary to give the following *elucidation*, of what must have appeared so clear and satisfactory to the meanest capacity. "The arterial system, on account of its *dentritic* expansion, resembles *positive electricity;* but the electricity disappears in the *indifferent* capillary vessels and in the absorbents—that is, in the reproductive system, and *yields* to simple attraction—magnetism." *Edin. Journal.*

Rochlaub's Theory almost blinds us phlegmatic Englishmen with its brilliancy. According to this ingenious gentleman, in inflammation, "*The fiery life plays into the material corporality, and endeavours to form a proper body to itself.*" (Ed. Journ.) In H. A. Goden's opinion, however, "the inflammatory nature consists in the fierceness, in the unbounded *egotism* of the *irritable moment.*" (Ibidem). I am, Gentlemen,

Your obedient servant,

Philo-Mysticus.

London, March 20, 1817.

*New Remedy for Tetanus.**—"Tetanus is a disease very common among the Tonga people; but still more common among the natives of the Fiji islands, who from their warlike habits are more frequently in the way of it: they adopt, however, a remedy which the Tonga people have borrowed of them, and consists in the operation of *tocolosi*, or passing a reed, first wetted with saliva, into the urethra, so as to occasion a considerable irritation, and discharge of blood; and if the general spasm is very violent, they make a seton of this passage, by passing down a double thread, looped over the end of the reed; and when it is felt in the perinæum, they cut down upon it, seize hold of the thread, and withdraw the reed, so that the two ends of the thread hang from the orifice of the urethra, and the doubled part from the artificial opening in the perinæum; the thread is occasionally drawn backwards and forwards, which excites very great pain and abundant discharge of blood. The latter operation Mr. Mariner has seen performed several times; but only twice for tetanus, arising in both instances from wounds in the foot: in these cases the spasms, but particularly the convulsive paroxysms, were exceedingly violent, extending to the whole body, neck, face, trunk, and extremities; but in neither case was the jaw permanently locked, though on every occasion it was violently closed for a few seconds. In both these cases the disease came on suddenly, three or four days after the wound was received, which was from an arrow not barbed. The moment the symptoms became evident, *tocolosi* was performed. In the short space of two hours, one of them was greatly relieved, and the other in about six or eight hours. The following day, one was quite well, and afterwards had no other attack; consequently the thread was withdrawn: but the other on the second day was not quite free from spasmodic symptoms, and a paroxysm coming on, the seton was moved frequently, which in two or three hours gave him great relief, and afterwards he had no other attack; it was thought prudent, however, to keep in the seton till the fourth or fifth day, when it was withdrawn. The effect of this operation was a considerable pain and tumefaction of the whole penis, but which gradually subsided in about five or six days: the artificial openings in both cases healed spontaneously, without any difficulty. From what Mr. Mariner has heard and seen of the success of this operation, at the Tonga islands, he is disposed to believe, that about three or four in ten recover by the aid of it."

*Entero-Mesenteric Fever.*—Dr. M. A. Petit, one of the physicians of L'Hotel-Dieu, has published a small treatise on a disease,

---

* Extracted from "An Account of the Natives of the Tonga Islands in the South Pacific Ocean, compiled and arranged from the extensive communications of Mr. Wm. Mariner, several years resident in those islands. By John Martin, M.D."

which he designates by the appellation of "*Fievre Entero-Mesenterique*," as it appeared in the said hospital in the years 1811, 12, and 13, from which we have extracted the following particulars:

This disease was, in general, incident to young men badly fed, and lately arrived in the capital. There were, however, several exceptions to this rule. Two students, for instance, whose circumstances were easy, and several others whose means were confined, suffered under this complaint. It affected all temperaments, but was most fatal among those of debilitated and exhausted constitutions. It was not confined to any particular season of the year; but it was both more frequent and mortal in a moist and cold, than in a warm and dry atmosphere. Previously to the reception of patients in the hospital, they had, for the most part, been affected with a sense of weakness, want of appetite, irregular fever, and frequently a degree of cholera. These symptoms gradually becoming worse, they were forced to desist from their avocations; and if the complaint was left to take its course, the progress was slow; but if any active medicines were exhibited, whether of the emetic or purgative kind, or if the patient had indulged in too much food or drink, then the march of the disease was greatly accelerated. The following is a portrait of the entero-mesenteric fever, as it first presented itself, in general, at the Hotel-Dieu.

The patient's countenance was indicative of dejection and despondency; the eye dull; the complexion faded, and rather livid, particularly about the lips and alæ nasi. The patient lies on his back, with disinclination to move. The skin appears remarkably rough and dry. There is torpor of the mental faculties; the answers to questions are slow but correct. There is little or no fever through the day; but towards evening it becomes developed, and in the night well marked. These nocturnal accessions of fever are not ushered in by any rigor, or sudden augmentation of heat; but they are accompanied by a turgescence of the vessels of the eye, and most commonly *delirium mite;* urgent thirst; dry mouth and loaded tongue. The alvine dejections are of a liquid bilio-serous nature (liquide bilio-sereux), very variable in frequency and quantity; but never so profuse as to account for the prostration of strength. The abdomen is soft, not swelled out (nullement metèorisé), and painful only on pressure, particularly in the lower part of the right side. If the hand be pressed pretty forcibly between the spine of the ileum and umbilicus, the patient will cry out, while spasmodic retractions of the lips and alæ nasi, and a general expression of pain in the countenance, will be visible. This symptom alone has frequently pointed out the disease before a question was asked; and in all cases, it was an important indication in the diagnosis.

This is a picture of the entero-mesenteric fever, in a mild degree, and in its most simple form; and as such, it yielded, without much difficulty, to remedial means, particularly when the latter were aided by a mild and dry state of the atmosphere. But where the remedies had not been early or judiciously applied, or where the patient was received at a more advanced period of the disease, then we had a much more formidable enemy to encounter.

In this case, the expression of debility and despondency was more marked. The complexion was more faded and earthy; the eye dull, sunk, and its vessels injected; somnolency and delirium constant; replies to questions more slow, but still correct; skin dry, harsh, and sometimes covered with petechiæ; subsultus tendinum; fever constant, but exacerbated with the other symptoms in the evening; pulse frequent, weak, easily compressed; tongue covered with a dry, brown crust; thirst excessive; belly more painful on pressure: pain sometimes confined to the lower part of the right side, without swelling; sometimes more diffused with fulness and tension of the parts: alvine dejections watery, fœtid, in general frequent, but sometimes the contrary; urine scanty; all excoriations of the skin tending to gangrene.

When the disease had come to this state, the issue was very uncertain, sometimes fatal, sometimes fortunate, especially when the temperature of the air was favourable. In the latter case, a gradual amelioration of the symptoms became perceptible; the physiognomy became more lively; the eye more brilliant; the answers more ready; the delirium remittent in the day, and ultimately disappearing; also the fever. The tenderness of the abdomen abated; the stools became less frequent and more natural; appetite returned early and vigorously, in a very remarkable and peculiar manner. These encouraging symptoms were accompanied by critical evacuations from the kidnies and skin, which greatly accelerated convalescence. The fatal termination was preceded and prognosticated by symptoms the reverse of the above.

*Post mortem Appearances.* The bodies evinced an unusual degree of putrefaction for the length of time between dissolution and dissection. The brain, lungs, and heart, were such as are usually found after death in putrid fevers (fievres adynamiques). The abdomen appeared, at first sight, in a natural state. The alimentary canal presented nothing unusual until we arrived at the middle of the ileum. There we began to perceive, on the exterior surface of the intestine, oval spots of a dark colour. Their number and dimensions increased as we approached the cæcum, below which it was rare to meet any of them. The coats of the intestine, occupied by these patches, were thicker than in any other place. The interior of the canal, from the stomach to the middle of the ileum, presented no morbid appearance. Below this, and exactly corresponding with the spots before described, were elliptic, well defined patches, formed by slight elevations of the mucous membrane of the intestines, and increasing in size and number, as we descended to the caput coli, where they disappeared. Besides these, we frequently found, in numerous instances, isolated pustules, scattered here and there over the internal surface of that part of the alimentary canal already alluded to, and which appeared to be of a similar nature with the spots above mentioned, but in a more advanced stage. The mesenteric glands were enlarged, some of them to the size of a nut, and quite changed, both in colour and structure, from a natural state.

*"Traité de la Fievre Entero-Mesenterique, &c."*

---

### *On Cerebral Inflammations, by M. Lespagnol.*

( Concluded from p. 263. )

CASE 2. A shoe-black boy, 13 years of age, of a naturally robust constitution, but debilitated for some time past by privations of every kind, was attacked, on the 25th May, with cephalalgia, nausea, vomiting, drowsiness, fever, and mild delirium; with cough, but no expectoration. In this state, he continued till the 31st, when he exhibited symptoms of the following kind: viz. dark red, earthy countenance; black crust on the lips and tongue; stupor; burning skin; vertigo. Four leeches to the neck; lemonade and nitre for drink; pediluvium with mustard in the evening. The stupor with delirium increased in the night. 1st of June, omnia exacerbata; respiration very quick. The same treatment continued. The delirium during the day, changed to profound coma in the night. Sinapisms to the feet. Decoction of cinchona internally. The rattles in the throat in the night; eyes insensible to light; grinding of the teeth in convulsions. 2d. Every spmptom, if possible, worse; foams at the mouth; face swelled and livid; action of the heart and lungs tumultuous. In the night, a general tetanic stiffness, with copious sweat. (roideur tetanique, generale, et une sueur copieuse). 3d. The symptoms less intense, but the patient no better. It was insisted on that tonics, diffusible stimuli, and derivatives to the surface (derivatifs a l'exterieur) should be employed. Shortly afterwards, the lips became brown; the tongue parched and hard; the pulse irregular and feeble; the belly flaccid; the insensibility general. The patient was relieved by the hand of death.

*Sectio Cadaveris.* Vessels of the brain injected; infiltration of the arachnoide tissue. From four to six ounces of serum at the basis cranii, and in the ventricles. A reddish tint in the cortical substance of the brain. Posterior lobe of the left lung converted into a substance like liver. (Hepatisation du lobe, &c.) Intestines inflamed in several places. A ball of ascarides in the small intestines. Enlargement of the mesenteric glands.

To the enlightened British practitioner, these cases cannot prove uninteresting. They place in a strong light the fatal practice of our Gallic brethren, whether that practice be of the passive or active kind. When *passive*, the inflammations and congestions were allowed to proceed with unmolested march — when *active*, the remedies were worse than the disease! Tonics and diffusible stimuli, when the vessels of the brain were so gorged, that nothing bu serous effusion prevented rupture of their coats ; when the lungs were hepatising, and the intestines inflaming ! May we not be proud of the able, the energetic, the successful measures which we employ in this country, by which so many thousand precious lives are saved to the community annually, and restored to their anxious parents?

M. Lespagnol next relates a case, in which the organic derangments in the digestive organs were of far greater extent. But as the inflammatory action made a very slow progress in the beginning, the sensorium was so gradually affected, that the progressive changes could be distinctly traced. Serous effusion in the ventricles put a period to life. Chronic inflammation, from the ravages committed on the stomach and intestines, must have been established there for a very long time.

Here M. Lespagnol quotes the authority of Cheyne, whose work appears only to have been analyzed in March, 1816, in one of the Paris Journals. From a number of facts analogous to the foregoing, our author draws his conclusions relative to the distinguishing marks of this dangerous complication of two distant inflammations ; of the causes which produce or augment it; of the prognosis, and the treatment.

The general symptoms of gastro-cerebral inflammation are, in the first days of the complaint, sense of weakness ; anorexy ; head-ache ; nausea ; vomiting; epigastralgia ; bitter taste in the mouth ; diarrhœa or constipation ; yellow tint in the countenance; fever with evening exacerbations ; occasional delirium, or somnolency. These symptoms aggravated by vomits. Afterwards, the face becomes of a dark red; the alæ nasi and lips of a yellow colour ; the eyes dull, watery, and inflamed ; slight convulsive motions in the muscles of the face ; tongue red at the edges ; grey or yellow near the root ; thirst urgent ; abdomen more or less painful; constipation more frequent than the contrary state; urine deep but clear, depositing afterwards a greyish sediment ; burning skin; pulse frequent, and pretty strong ; respiration in proportion; pain often felt in the right hypochondrium ;guttural angina; dull pains in the limbs.

If, in this state of things, we treat the disease with emetics, tonics, or anthelmintics, the progress will be accelerated, and an alarming train of symptoms will supervene ; as profound stupor ; violent delirium ; livor of the countenance ; parched state of the lips and tongue, with a black crust ; vomiting of every thing drunk ; cephalalgia and abdominal pain ceasing and returning ; pulse small, quick, irregular, and intermittent , intense heat of skin : insensibility supervenes, and becomes general ; while trismus, or tetanic stiffness of the neck or some of the limbs, makes its appearance. The respiration now becomes laborious ; the bladder is paralyzed ; and after two or three days duration of these morbid phænomena, death is ushered in, amidst convulsions.

The progress of this disease is rapid, and its termination in recovery or death usually takes place in from eight to fifteen days. Dissection constantly shews inflammation, more or less intense, of the stomach and intestines; and a corresponding morbid change of structure in the brain or its investing membranes. The prognosis must be founded on the state of the disease, the intensity of the symptoms, the peculiarity of the constitution, and the irritability of the subject; also on the nature and obstinacy of the causes which keep up the irritation in the digestive organs.

Although this pathology is perhaps correct in the outline, we think it is defective in many important points.

The *hepatic functions* are not sufficiently kept in view. The primary symptoms are evidently not those of *inflammation*. Torpor and deficient action in the biliary organ are, in these cases, almost universally the first links in the chain of morbid phænomena, and exercise a powerful influence over the progressive stages of the disease. This is not a mere hypothesis; it is a practical fact, and upon it hinges the most important measure in the treatment, as will be presently seen.

*M. Lespagnol's Methodus Medendi.*—"In the first days of the complaint we must confine ourselves to the exhibition of dilute acidulous drinks; very low regimen; emollient applications to the abdomen; avoiding vomits, which are always pernicious. About the 4th or 5th day, if the symptoms become more severe, we must have recourse to a more active antiphlogistic treatment. Leeches may be applied to the abdomen or anus; and when cerebral congestion is unequivocally demonstrated, we must place all reliance on derivatives, as warm pediluvia; blisters and sinapisms to the lower extremities; leeches in the track of the jugular veins. As for the rest, I conceive that the case is beyond the power of art *after* effusion has once taken place in the brain."

Whatever truth there may be in the last sentence, we conceive, that the *methodus medendi* is most lamentably devoid of energy. When the disease is, as it were, in its *cradle*, we quietly sit by, feeding it with acidulous drinks, and only attack it vigorously when it has acquired a gigantic force that must too frequently baffle all our skill! In those four days, the biliary apparatus should, we conceive, be roused into healthy action by mercurial purgatives, which would at the same time, clear the intestinal track of a mass of irritating morbid secretions. The portal and mesenteric circulations should be kept free by alteratives—by the warm bath (the best of all derivatives) and cathartics. To the local abstractions of blood from the head, the abdomen, or the anus, we do not object; but experience has taught us that they will seldom be necessary, where due attention is paid to clearing daily away all morbid secretions from the hepatic, gastric, and intestinal apparatus.

The Medical Society of London celebrated their 44th Anniversary on the 8th of March last, which was respectably attended. The Scrutators reported that the Election for Officers had fallen upon the following gentlemen: viz.

PRESIDENT,—Dr. WALSHMAN.
VICE PRESIDENTS,
Drs. Temple, Merriman; John Abernethy, George Young, Esqrs.
TREASURER,—JOHN ANDREE, Esq.
LIBRARIAN,—Dr. HANCOCK.
SECRETARY,—H. BLEGBOROUGH, Esq.
REGISTRAR,—T. J. PETTIGREW, Esq.
ORATOR for 1818, Dr. UWINS.

COUNCIL:—Drs. Adams, Babington, Clutterbuck, Hamilton, Laird, Pinckard, Uwins. Messrs. Norris, E. Leese, B. Atkinson, T. J. Pettigrew, Mathias, Taunton, Damant, Bartlett, Johnston, Sutleffe, Stevenson, E. Austin, Edwards, Hooper, Beveridge, Wachsell, Heath, Vance, Whateley, R. Adams, R. James, and H. C. Field.

The report of the finances and improvement in the library was very favourable.—The Second Part of the 1st volume of the Transactions was announced to be ready for delivery to the Fellows.—The Oration, " on the Value and Importance of the Medical Profession, was delivered by Mr. STEVENSON; of which an abstract will be given in our next Number.

---

Dr. MERRIMAN and Dr. LEY will re-commence their Lectures on Midwifery and the Diseases of Women and Children at the *Middlesex Hospital*, on Monday, April the 21st, at half past ten o'clock.

Dr. BURROWS has just published a work, entitled, " Cursory Remarks on a Bill, now in the House of Peers, For regulating of Mad-Houses; its probable Influence upon the Physical and Moral Condition of the Insane; and upon the Interests of those concerned in their Care and Management."

Speedily will be published, A Medico-Chirurgical and Biographical Chart of Medical Science, from Hippocrates to the present Time; exhibiting, in a condensed Form, the Progress and present State of that Science; with short Biographical Notices of the most eminent Authors, in this and other countries, who have contributed to enhance our Stock of Medical Knowledge.

DEATHS.—At his house in Vernon Place, Bloomsbury, in his 74th year, Charles Combe, M. D. F. R. S. and S. A.—At Edinburgh, of a typhus fever, caught in his attendance on a poor family, Mr. John Fyfe, Surgeon, son of Mr. Andrew Fyfe, the well-known Demonstrator in the Anatomical Class in that University.—In Tottenham Court Road, aged 63, Mr. Arthur Freake, Apothecary.

